# Casting vs Surgical Treatment of Children With Medial Epicondyle Fractures: A Randomized Clinical Trial

**DOI:** 10.1001/jamanetworkopen.2025.8479

**Published:** 2025-05-06

**Authors:** Petra Grahn, Ilkka Helenius, Tero Hämäläinen, Reetta Kivisaari, Yrjänä Nietosvaara, Juha-Jaakko Sinikumpu, Jenni Jalkanen, Eliisa Löyttyniemi, Matti Ahonen

**Affiliations:** 1 Department of Pediatric Orthopedics and Traumatology, New Children’s Hospital, Helsinki University Hospital, University of Helsinki, Helsinki, Finland; 2 Department of Orthopaedics and Traumatology, University of Helsinki and Helsinki University Hospital, Helsinki, Finland; 3 Department of Pediatric Surgery, Orthopaedics, and Traumatology, University of Turku and Turku University Hospital, Turku, Finland; 4 Department of Radiology, HUS Diagnostic Center, University of Helsinki and Helsinki University Hospital, Helsinki, Finland; 5 Department of Pediatric Surgery, Kuopio University Hospital, University of Eastern Finland, Kuopio; 6 Department of Pediatric Surgery, Orthopaedics, and Traumatology, University of Oulu and Oulu University Hospital, Oulu, Finland; 7 Research Unit of Clinical Medicine, University of Oulu and Oulu University Hospital, Oulu, Finland; 8 Department of Biostatistics, University of Turku and Turku University Hospital, Turku, Finland

## Abstract

**Importance:**

Displaced pediatric medial humeral epicondyle fractures are traditionally treated nonoperatively with casting. However, the use of surgical treatment has increased despite limited high-level evidence supporting its benefits.

**Objective:**

To determine whether open surgical reduction and internal fixation improve functional outcomes compared with long arm casting in children with displaced medial humeral epicondyle fractures at 12 months post injury.

**Design, Setting, and Participants:**

This noninferiority randomized clinical trial was conducted in 4 university hospitals in Finland between August 30, 2019, and August 22, 2023, with a 12-month follow-up completed August 20, 2024. Participants included children (aged 7-16 years) with nonincarcerated medial humeral epicondyle fractures and more than 2 mm of displacement. Data analysis was based on intention to treat.

**Interventions:**

Open reduction and fixation, followed by a long arm cast for 4 weeks, or long arm cast without reduction for 4 weeks.

**Main Outcome and Measure:**

The primary outcome was the Quick Disabilities of the Arm, Shoulder, and Hand (QDASH) score at 12 months (range, 0-100 points, with 0 denoting no disability and 100 extreme disability; prespecified noninferiority margin was 6.8 points).

**Results:**

Seventy-two patients were randomized (43 [59.7%] female; mean [SD] age, 12.1 [2.1] years; range, 7.9-15.9 years), with 37 (19 [51.4%] female) to the surgery group (mean [SD] age, 12.2 [2.3] years; range, 7.9-15.9 years) and 35 (24 [68.6%] female) to the cast group (mean [SD] age, 11.9 [2.0] years; range 7.9-15.9 years). At 12 months, the mean QDASH score was 1.73 (95% CI, 0.65-2.81) in the surgery group and 2.71 (95% CI, 0.52-4.90) in the cast group, showing noninferiority (mean difference, −0.98 [95% CI, −2.95 to 0.98] points). The cosmetic visual analog scale favored the cast group, with a statistically significant between-group difference of −8.9 points (95% CI, −16.6 to −1.2 points; *P* < .001). Nonunion occurred in 1 of 37 surgically treated patients (2.7%) and 24 of 35 cast-treated patients (68.6%). No crossovers from casting to surgery occurred.

**Conclusions and Relevance:**

In this randomized clinical trial of displaced medial epicondyle fractures, treatment with casting alone was noninferior at 12 months to surgical reduction and internal fixation followed by casting. Findings support nonoperative care as effective at 1 year; longer-term outcomes remain to be studied.

**Trial Registration:**

ClinicalTrials.gov Identifier: NCT04531085

## Key Points

**Question:**

Does cast immobilization provide similar clinical outcomes compared with surgical treatment for displaced pediatric medial epicondyle fractures?

**Findings:**

In this randomized clinical trial involving 72 pediatric patients, casting only was noninferior to surgery as measured with the Quick Disabilities of Arm, Shoulder, and Hand score. At the primary end point of 12 months, the mean score in the surgery group was 1.7 compared with 2.7 in the cast group, with the difference not reaching the noninferiority margin of 6.8.

**Meaning:**

These findings suggest that cast immobilization is a viable alternative to surgery for treating displaced pediatric medial epicondyle fractures, offering comparable functional results.

## Teaser

This randomized clinical trial assesses whether casting without reduction is noninferior to open surgical reduction and internal fixation for treatment of displaced medial epicondyle fractures in pediatric patients.

## Introduction

Two-thirds of all pediatric fractures occur in the upper extremity.^1^ The surgical treatment of these fractures has increased rapidly with the development of pediatric internal fixation devices.^2^ However, there is a paucity of high-level evidence to support this practice, and to our knowledge, no randomized clinical trials have been published comparing nonoperative with operative treatment of pediatric upper extremity fractures.

Medial humeral epicondyle fractures, which typically result from a fall on an outstretched hand, have an incidence of 3 or greater per 100 000 children and account for 12% to 20% of all pediatric elbow fractures.^3^ These fractures are associated with elbow dislocation in 30% to 50% of cases, and ulnar nerve dysfunction is observed in 10% to 16% of cases.^4^ Nondisplaced medial epicondyle fractures are commonly treated with casting, while traditionally, even displaced fractures have often been managed with cast immobilization.^5,6,7^ A long-term study of children with a displaced medial epicondyle fracture with a mean follow-up of 35 years^8^ reported that 12 of 56 patients (21.4%) had mild to moderate symptoms, although none interfered with activities or work. Despite the high rate of nonunion with conservative treatment of isolated displaced medial epicondyle fractures, radiological union has shown poor correlation with functional outcomes.^9,10^

There is currently a lack of consensus on the treatment of displaced medial epicondyle fractures in children, except in cases where the fragment is trapped in the elbow joint, for which surgical intervention is generally recommended.^11^ Surgical reduction and fixation of displaced fractures may increase the likelihood of bony union and potentially improve elbow stability and function due to the attachment of the medial ulnar collateral ligament to the fracture fragment.^12,13^ This stability could allow for an earlier return to sports.^14,15^

Two systematic reviews^10,16^ have examined the optimal treatment for these injuries. One concluded that both nonsurgical and surgical treatments yield similar outcomes,^10^ while the other recommended surgical fixation to achieve fracture union and maximum elbow stability.^16^ This controversy has resulted in varied treatment practices and identified research on the optimal management of these fractures as a high priority for randomized clinical trials in pediatric fractures.^11^ This noninferiority randomized clinical trial investigates the outcomes of open surgical reduction and internal fixation compared with casting without reduction in the treatment of displaced pediatric medial epicondyle fractures.

## Methods

This multicenter, parallel group, noninferiority, nonblinded randomized clinical trial compared operative vs nonoperative treatment of pediatric medial epicondyle fractures with more than 2 mm of displacement, excluding cases with joint incarceration or ulnar nerve dysfunction. The trial was approved by the Institutional Review Board of the Helsinki and Uusimaa Hospital District. The full trial protocol is available in Supplement 1 and has been published previously.^17^ This study was reported according to the Consolidated Standards of Reporting Trials (CONSORT) reporting guideline and was monitored according to the trial data monitoring protocol provided by the Clinical Research Institute HUS, Helsinki, Finland. Written informed consent was obtained from guardians, with child-appropriate versions provided per Finnish guidelines.

### Participants

**Figure 1.**
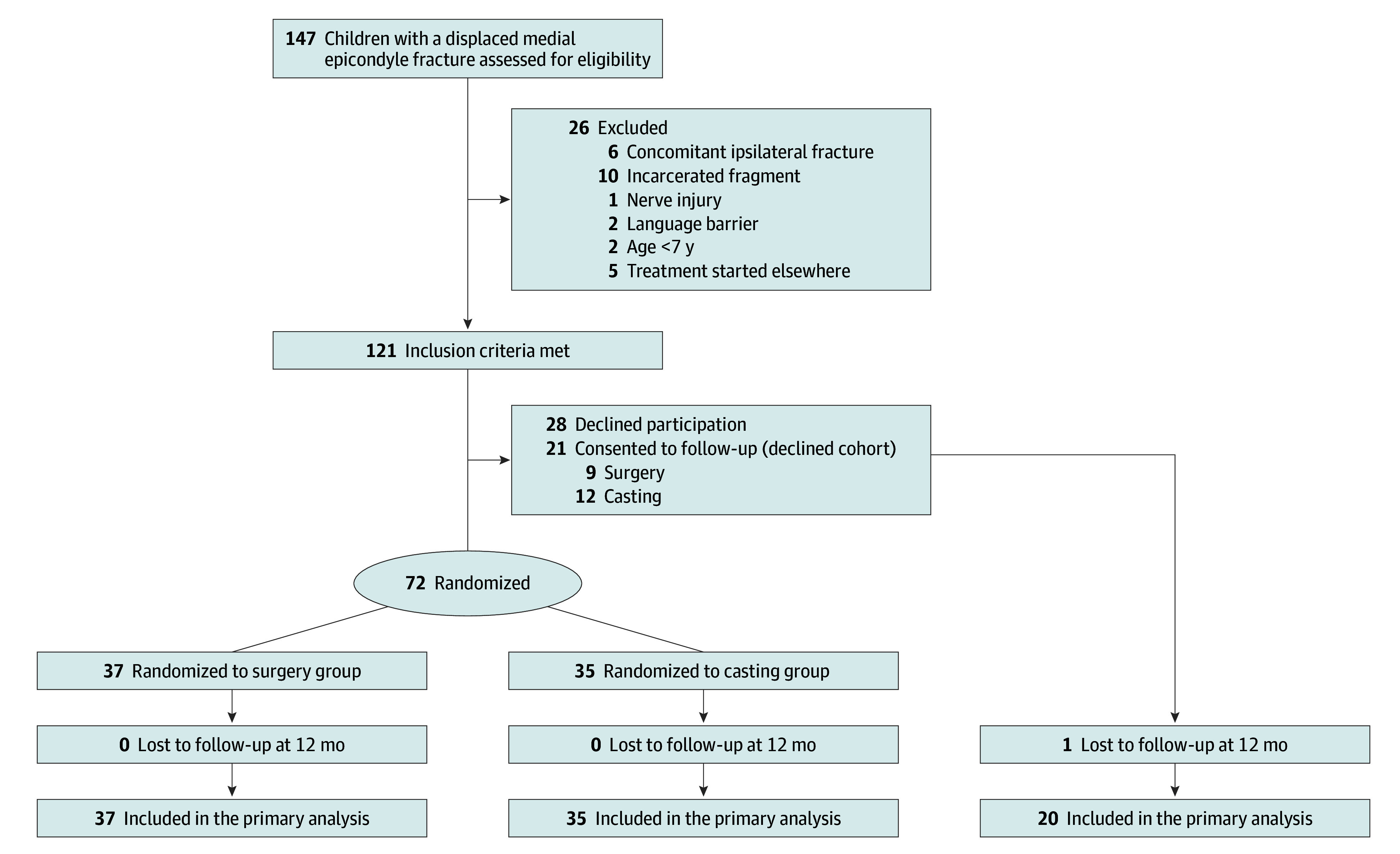
Enrollment, Randomization, and Analysis of Patients in the Trial Patients who declined randomization were invited to participate in an observational cohort (declined cohort) that followed the study protocol and were treated according to their preference. The declined cohort was analyzed separately from the randomized cohort. All patients who reached the final follow-up of 12 months were included in the analysis.

Participants were recruited from 4 university hospitals in Finland (Helsinki, Kuopio, Oulu, and Turku) (Figure 1). Consecutive patients aged 7 to 16 years presenting to the emergency department with a medial epicondyle fracture were screened for eligibility by a consultant orthopedic surgeon (P.G., I.H., T.H., Y.N., J.-J.S., J.J., and M.A.). Recruitment occurred from August 30, 2019, to August 22, 2023, and 12-month follow-up was completed on August 20, 2024. Inclusion criteria were fractures with at least 2 mm of displacement, excluding cases with joint incarceration, ulnar nerve dysfunction, pathological or open fractures, systemic bone disease, concomitant upper limb fractures requiring surgery, or conditions precluding full participation in follow-up (eTable 1 in Supplement 2). Patients with elbow dislocation were included if postreduction radiographs met the inclusion criteria.

### Baseline Assessments

Baseline data included patient demographics, injury mechanism, side of injury, dominant hand, and motor or sensory function assessments. Standard anteroposterior and lateral elbow radiographs were obtained following reduction of any dislocation, and computed tomographic scans assessed fracture extent. Fracture displacement was measured on radiographs and computed tomographic scans as described previously.^18^

### Randomization

Participants were randomly assigned (1:1) to operative or nonoperative treatment using a block size of 10. The randomization was performed using the Research Randomizer, version 4.0,^19^ with allocations concealed in sequentially numbered, opaque envelopes prepared by a research nurse uninvolved in trial participant care. Randomization was stratified by center. Envelopes were securely stored at each center, with allocation determined upon opening. The allocation sequence was maintained at the primary center (New Children’s Hospital) and concealed from recruiting physicians. In the trial, patient allocation was determined when the assigned envelope was opened.

### Interventions

In the surgery group, the procedure was scheduled within 7 days of injury. Fixation involved open reduction with a 4.0-mm screw (with or without a washer), Kirschner wires, or a bone anchor for internal fixation (eTable 2 in Supplement 2). A long arm cast with the elbow at 90° of flexion and the forearm in neutral prosupination was applied for 4 weeks (eAppendix 2 in Supplement 2). In the casting group, a long arm cast was applied for 4 weeks with the elbow at 90° of flexion and the forearm in neutral prosupination.

Documentation included injury-to-initiation of treatment time, fixation method, and surgeon’s expertise. All patients followed a standardized exercise regimen (eAppendix 3 in Supplement 2), with physiotherapy offered if no improvement in elbow range of motion was observed within 2 weeks of cast removal. A parallel nonrandomized patient preference arm was included initially but discontinued on March 16, 2020, due to the COVID-19 pandemic.

### Outcomes

The primary outcome was the Quick Disabilities of the Arm, Shoulder and Hand (QDASH) score at 12 months.^20^ The QDASH score ranges from 0 to 100 points, with 0 denoting no disability and 100, extreme disability. Secondary outcomes included active elbow range of motion, carrying angle, valgus stress test, moving valgus test, grip strength, ulnar nerve sensory and motor dysfunction, cold sensitivity, scores on the Pediatric Quality of Life Inventory Generic Core Scale (PedsQL) and PedsQL Pain Questionnaire, Mayo Elbow Performance Score (MEPS), cosmetic visual analog scale (VAS), fracture union rates, return to sports, additional procedures, and crossover between treatment groups.^21,22,23,24,25^ The PedsQL score, its physical health subscore, the MEPS, and the cosmetic VAS are similarly calculated from 0 to 100, with a higher score representing a better outcome. The PedsQL Pain scores are calculated from 0 to 100, with 0 indicating no disability or pain and 100 indicating the highest level of disability or pain. Assessments were scheduled at 1, 3, 6, and 12 months.

### Blinding

The recruiter and biostatistician (E.L.) were blinded to group assignments. Follow-up data were collected by physicians uninvolved in the trial to ensure blinding.

### Sample Size and Noninferiority Margin

Based on previous studies, a noninferiority margin of 6.8 points on the QDASH score was used.^26,27^ With an SD of 10 points, a .05 significance level, and 80% power, 27 patients per group were required. Allowing for a 10% dropout rate, the target sample size was 30 patients per group as published in the study protocol.^25^

Initially, our protocol specified a target enrollment of 120 patients to facilitate a subgroup analysis based on age.^17^ However, we were unable to recruit the full sample within the permitted 5-year study period, and enrollment concluded with 72 participants. This sample size was sufficient to achieve the primary objective of comparing operative and nonoperative treatments but limited our ability to perform the planned age-based subgroup analysis.

### Declined Cohort

Patients who declined randomization were invited to participate in an observational cohort (declined cohort) that followed the study protocol and were treated according to their preference. The declined cohort was analyzed separately from the randomized cohort. All patients who reached the final follow-up of 12 months were included in the analysis.

### Statistical Analysis

Continuous variables were summarized using means and SDs for normally distributed data or medians with lower and upper quartiles (IQR) for skewed data. Categorical variables were presented as counts and percentages. The primary and secondary outcomes were reported and compared using the intention to treat principle. The primary outcome, the QDASH score at 12 months, was analyzed using linear mixed models for repeated measures. This model included data from all follow-up visits (1, 3, 6, and 12 months) and accounted for the correlation between repeated measures within participants.

To meet the assumption of normality for studentized residuals, the QDASH score was transformed using the natural logarithm. The model used an unstructured covariance structure, which provided the best fit for the data, and the Kenward-Roger correction was applied to adjust the degrees of freedom. Group differences were estimated at the 12-month follow-up, with reported *P* values derived from this model. All estimated means in the models were then back transformed (exponentiated) to the original scale to facilitate clinical evaluation of the results. Given the study’s noninferiority design, the primary analysis aimed to assess whether the mean difference in QDASH scores between groups at 12 months was within the prespecified noninferiority margin of 6.8 points. In addition to the transformed analysis, a linear mixed model without logarithmic transformation was used to calculate a 95% CI for the mean difference in QDASH scores, confirming noninferiority if the upper limit of the CI did not exceed 6.8 points. An estimation of the median group difference was not feasible as both groups had a median of zero.

The same linear mixed model approach was applied to secondary continuous outcomes, including active elbow range of motion, extension deficit, and PedsQL scores. For PedsQL scores, a transformation was applied to adjust for left-skewed distribution (most children having high quality-of-life scores) by creating a mirror distribution (101 – score) and then calculating the square root. Secondary outcomes that did not meet assumptions for parametric analysis (eg, grip strength, carrying angle, and VAS scores) were analyzed using the Wilcoxon rank sum test at each follow-up visit. Associations between group and moving valgus test, valgus stress test, and additional procedures were evaluated with Fisher exact test. In most cases, CI calculations for difference of medians between the groups did not convert, and therefore they are not displayed. Two-tailed *P* < .05 indicated statistical significance unless otherwise stated. All analyses were conducted using SAS software, version 9.4 (SAS Institute Inc). Data from the declined cohort were analyzed separately from the randomized using the same methods.

## Results

### Characteristics of the Patients

Among the 121 patients who fulfilled the inclusion criteria, 72 patients were randomized (43 [59.7%] female and 29 [40.3%] male; mean [SD] age, 12.1 [2.1] years; range, 7.9-15.9 years), with 37 (19 [51.4%] female and 18 [48.6%] male) assigned to the surgery group and 35 (24 [68.6%] female and 11 [31.4%] male) to the casting group. The mean (SD) age was 12.2 (2.3) years (range, 7.9-15.9 years) in the surgery group and 11.9 (2.0) years (range, 7.9-15.9 years) in the casting group. All patients received the treatment to which they were allocated. Baseline characteristics, including hand dominance, injury mechanism, and initial fracture displacement, were similar between groups (Table 1). No patients were lost to follow-up during the 12-month follow-up period.

**Table 1.** Baseline Demographic and Injury Characteristics

Characteristics	Patient group		
	Surgery (n = 37)	Casting (n = 35)	Declined cohort (n = 21)
Age at injury, mean (SD) [range], y	12.2 (2.3) [7.9-15.9]	11.9 (2.0) [7.9-15.9]	12.5 (2.2) [7-15.4]
Sex, No. (%)			
Female	19 (51.4)	24 (68.6)	12 (57.1)
Male	18 (48.6)	11 (31.4)	9 (42.9)
Dominant side injured, No. (%)	19 (51.4)	17 (48.6)	8 (38.1)
Injury mechanism, No. (%)			
Gymnastics^a^	22 (59.5)	24 (68.6)	12 (57.1)
Arm wrestling or wrestling	3 (8.1)	3 (8.6)	1 (4.8)
Fall from standing height^b^	7 (18.9)	5 (14.3)	4 (19.0)
Fall from height^c^	5 (13.5)	3 (8.6)	4 (19.0)
Elbow dislocation, No. (%)^d^	13 (35.1)	12 (34.3)	9 (42.9)
Primary fracture dislocation, mean (median) [range], mm^e^			
Anteroposterior radiograph	8.7 (2.6) [3.6-14.0]	8.9 (1.7) [6.0-13.5]	8.3 (2.6) [4.0-13.5]
Lateral radiograph^f^	11.8 (5.2) [6.5-21.5]	10.2 (2.9) [5.0-12.1]	9.8 (6.5) [1.0-18.0]
Coronal computed tomography	7.7 (2.4) [3.0-15.5]	8.0 (2.2) [2.5-14.0]	6.9 (2.8) [3.0-13.5]
Sagittal computed tomography	10.9 (2.6) [5.5-17.5]	11.1 (2.5) [9.5-16.5]	11.6 (3.5) [2.0-17.0]
Time from injury to treatment, mean (SD) [range], d	3.4 (2.2) [0-9.0]	1.5 (2.4) [0-8.0]	2.0 (1.9) [0-8.0]
Immobilization time, mean (SD) [range], wk	4.5 (0.6) [3.3-5.1]	4.4 (0.3) [3.6-5.1]	4.5 (0.5) [3.9-5.1]

^a^ Also includes cheerleading, parkour, bouldering, and dancing.

^b^ Also includes ball games (soccer and ice hockey).

^c^ Also includes biking, electric scooter, downhill skiing, and skateboarding.

^d^ Indicates radiographic complete elbow joint dislocation.

^e^ Displacement was measured from radiographs and computer tomography images as described by Edmonds.^18^

^f^ Fragment was not visible in the surgery group in 29, casting group in 25, and declined cohort in 15 patients.

### Primary Outcome

At 12 months, the mean QDASH score was 1.73 (95% CI, 0.65-2.81) in the surgery group and 2.71 (95% CI, 0.52-4.90) in the casting group (Table 2). Model-based mean difference between the groups was −0.98 (95% CI, −2.95 to 0.98) points (surgery minus casting) and, based on the CI, noninferiority criteria was met.

**Table 2.** Primary and Secondary Outcomes at 12 Months

Outcome	Surgery group (n = 37)		Casting group (n = 35)		*P* value
	Mean	Median (IQR)	Mean	Median (IQR)	
**Primary**					
QDASH score	1.73	0.0 (0.0 to 2.3)	2.71	0.0 (0.0 to 2.0)	.86
95% CI	0.65 to 2.81	NA	0.52 to 4.90	NA	
**Secondary**					
Clinical findings					
Elbow range of motion^a^	150.6	150.0 (145.0 to 157.0)	141.1	145.0 (140.0 to 155.0)	.27
Flexion deficit^b^	1.3	0.0 (0.0 to 0.0)	1.7	0.0 (0.0 to 0.0)	.54
Extension deficit^b^	2.4	0.0 (0.0 to 5.0)	3.0	0.0 (0.0 to 0.0)	.62
Carrying angle deficit^b^	−0.7	0.0 (0.0 to 0.0)	−0.8	0.0 (−1.0 to 0.0)	.42
Moving valgus test, No. (%)^c^	1 (2.7)	NA	4 (11.4)	NA	.19
Valgus stress test, No. (%)^c^	5 (13.5)	NA	5 (14.3)	NA	>.99
Grip strength, kg^d^	0.4	0.0 (−2.0 to 1.0)	1.1	0.0 (−0.3 to 2.0)	.15
QDASH module score					
Sports or performing arts	1.5	0.0 (0.0 to 0.0)	2.9	0.0 (0.0 to 0.0)	.32
PedsQL^e^					
Total score	94.5	97.0 (92.4 to 100.0)	92.9	96.7 (87.4 to 100.0)	.34
Physical function score	96.2	100.0 (97.0 to 100.0)	95.0	97.0 (91.0 to 100.0)	.26
PedsQL Pain Questionnaire^f^					
Current pain	1.1	0.0 (0.0 to 1.1)	1.7	0.0 (0.0 to 0.0)	.27
Worst pain in last 7 d	3.8	0.0 (0.0 to 4.0)	4.4	0.0 (0.0 to 2.0)	.22
Cosmetic VAS^g^	77.4	91.0 (76.6 to 98.0)	94.6	100 (90.1 to 100.0)	<.001
MEPS^h^	97.6	100.0 (100.00 to 100.0)	98.0	100.0 (100.0 to 100.0)	.59
Additional procedures, No. (%)^i^	4 (10.8)	NA	0	NA	.12

Abbreviations: MEPS, Mayo Elbow Performance Score; NA, not applicable; PedsQL, Pediatric Quality of Life Inventory Generic Core Scale; QDASH, Quick Disabilities of Arm, Shoulder and Hand; VAS, visual analog scale.

^a^ Measured by goniometer and reported as the difference in degrees between full flexion and extension.

^b^ Compared with the uninjured side and reported as difference in degrees.

^c^ Number of patients with instability compared with the uninjured side. Surgery group: 3 pain, 2 looseness to uninjured side. Cast group: 2 pain, 3 looseness to uninjured side.

^d^ Measured with a dynamometer and expressed as difference to uninjured side.

^e^ Indicates a brief measure of health-related quality of life in children and young people with a subscale describing physical function. Both are scored from 0 (worst possible) to 100 (best possible).

^f^ Ranges from 0 (no pain) to 100 (worst imaginable pain).

^g^ Ranges from 0 (worst possible appearance) to 100 (best possible appearance).

^h^ Composite score of 100 used to report elbow outcome with higher scores reflecting better results.

^i^ Any surgical procedures related to the injury after index management.

### Secondary Outcomes

**Figure 2.**
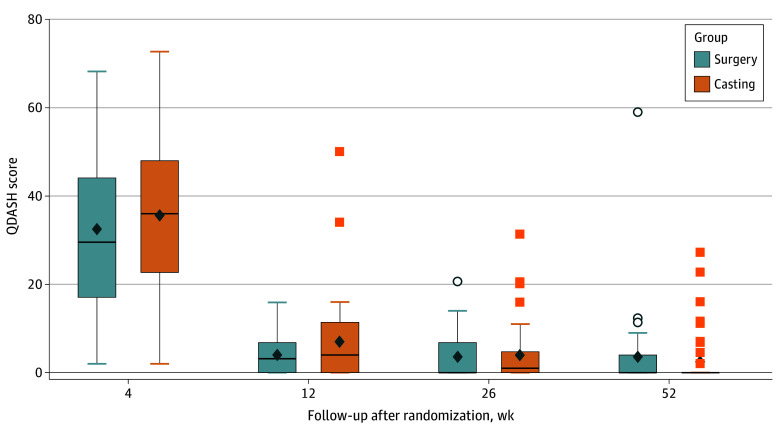
Quick Disabilities of Arm, Shoulder and Hand (QDASH) Score Over Time The QDASH scores range from 0 to 100 points, with 0 denoting no disability and 100, extreme disability. The boxes indicate the 25th and 75th percentiles (IQR) of the observed values, with horizontal lines inside the boxes representing the median QDASH scores. The vertical lines extending from the boxes show the highest and lowest values within 1.5 times the IQR. Data points beyond these error bars indicate individual values outside this range. Diamonds represent mean values.

There were no statistically significant differences in QDASH scores between the surgery and cast groups at 1, 3, or 6 months (Figure 2). There was a statistically significant difference in the active elbow range of motion between the groups favoring the surgery group at 3 months (mean, 135.2° [median, 140.0°; IQR, 130.0°-145.0°] vs 125.0° [median, 125.0°; IQR, 116.0°-140.0°]) and 6 months (mean, 144.1° [median, 145.5°; IQR, 137.5°-145.5°] vs mean 135.3˚[median 137.0˚; IQR 130˚-145˚) (eTables 5 and 6 in Supplement 2). The valgus stress test result was positive in 3 of 37 patients in the surgery group and 9 of 30 in the cast group at 6 months (*P* = .051) (eTable 6 in Supplement 2). Similarly, a statistically significant difference in grip strength was observed at 1 month favoring the surgery group (mean, 8.3 [median, 8.0; IQR, 6.0-10.0] vs 7.0 [median, 6.0; IQR, 4.5-7.9] kg compared with the uninjured side) (eTable 4 in Supplement 2) and in the cosmetic VAS at 6 months favoring the casting group (mean, 76.1 [median, 84.3; IQR, 70.9-94.8] vs 86.2 [median, 100.0; IQR, 85.0-100.0] mm) (eTable 6 in Supplement 2). No other secondary outcome measures detected any between-group differences at 1, 3, and 6 months.

At 12 months, the median cosmetic VAS score was 90.0 (95% CI, 86.8-100.0) mm for the surgery group compared with 100.0 (95% CI, 100.0-100.0) mm for the casting group, with a statistically significant difference of −8.9 (95% CI, −16.6 to −1.2) points (*P* < .001). At the 12-month final follow-up, there were no statistically significant differences between the groups in grip strength, range of motion, carrying angle, elbow stability, or ulnar nerve function. Neither was there any between group difference in PedsQL or PedsQL pain scores or MEPS scores (Table 2). None of the patients reported ulnar nerve dysfunction or cold intolerance.

All 65 patients who participated in competitive sports (30 in the surgery group and 30 in the casting group) or music (3 in the surgery group and 2 in the casting group) before the injury returned to their preinjury level or higher. There was no statistically significant difference in the time from injury to return to sports or music between the groups (median, 2.0 [95% CI, 1.7-3.6] months for the surgery group vs 2.0 [95% CI, 1.4-2.7] months for the casting group). Missing data at different time points are reported in eTable 3 in Supplement 2.

### Adverse Events and Crossover

Fracture nonunion was observed in 1 of 37 (2.7%) surgically treated patients by the 12-month follow-up and in 24 of 35 (68.6%) cast-treated patients. Four patients in the surgery group asked for screw removal at a mean (SD) of 8.9 (2.2) months post surgery (range, 6.3-12.2 months) due to screw-related irritation and pain. Two patients experienced diminished sensation postoperatively: one in the medial border of the forearm and the other in the palmar and pulp area of fingers 4 and 5. Both recovered fully by 6 months. No surgical site infections were reported.

By the 12-month follow-up, none of the 35 patients in the casting group required additional treatment or experienced symptoms of nerve injury or irritation. No adverse effects related to casting were noted in either group. No crossover from cast treatment to surgery occurred.

### Declined Cohort

Of the 49 eligible patients who declined randomization, 21 consented to follow-up (9 chose surgery and 12 opted for casting) (Figure 1). Demographic characteristics are given in eTable 7 in Supplement 2. At 12 months, after excluding 1 patient lost to follow-up, the QDASH score differed significantly between groups. The model-based mean scores were 5.6 (95% CI, 2.3-13.9) in the surgery group and 0.9 (95% CI, 0.4-1.8) in the casting group (*P* = .004).

## Discussion

This multicenter randomized clinical trial found that casting without reduction is noninferior to surgical reduction and internal fixation for displaced humeral medial epicondyle fractures in children at 12 months of follow-up. This investigation is, to our knowledge, the first randomized clinical trial comparing casting without reduction and open reduction and internal fixation in displaced pediatric medial humeral epicondyle fractures. Previously in nonrandomized comparative investigation, similar results for both treatments were found for nonoperative and operative treatment with a mean (SD) QDASH score of 1.3 (1.9) in the operative group and 0.1 (0.4) in the nonoperative group.^28^ In the present study, primary outcome evaluation found no difference in the median QDASH score at 1-year follow-up (1.73 vs 2.71).

Displaced medial epicondyle humeral fractures in children are commonly treated surgically to enable early range of motion, prevent elbow instability, and reduce the risk of fracture nonunion.^10,11,16^ However, previous observational studies on these fractures have reported conflicting results regarding the benefits of surgical intervention.^8,10^

Malunion of displaced medial epicondyle fractures can reduce grip strength, as the flexor muscles of the wrist and fingers originate from this area.^29^ Although grip strength at 4 weeks was better in the surgical group than in the casting group, the difference was below the smallest detectable difference for the Jamar dynamometer (2.05 kg for children and 2.16 kg for adolescents), and it had equalized by the 3-month follow-up.^30^

Both the trauma from the initial injury and subsequent open reduction and fixation, combined with immobilization in a long-arm cast, can affect the total range of motion of the elbow. Although there were differences in elbow range of motion between the groups at 3 and 6 months, these differences were within the SE of measurement (11.5°) for elbow goniometry.^31^ The medial collateral ligament attaches to the medial epicondyle and distal humeral region, raising concerns that an unfixed fracture could lead to valgus instability.^13^ A positive valgus stress test result, which indicates instability, is often considered a sign that open reduction and internal fixation may be necessary for displaced fractures.^1^ In the casting group, a higher percentage of positive valgus stress test results was found at the 6-month evaluation. Despite early concerns regarding valgus stability, no patient in the casting group exhibited clinical signs of posttraumatic elbow instability or required delayed surgical stabilization, and return-to-sports times were comparable between treatment groups. These findings suggest that nonoperative management does not lead to persistent instability or functional impairment at 1 year. However, longer-term studies are needed to determine whether subtle instability or late functional deficits emerge beyond the first year of follow-up.

Cosmetic appearance of the upper limb can significantly impact quality of life.^32^ Although the scar from open reduction and fixation of the medial epicondyle is small, it remains in a visible area. In this study, the cosmetic outcome favored casting alone, with the difference between groups increasing over time. By the final follow-up, this difference surpassed the minimal clinically important difference for the cosmetic VAS score (15 mm), suggesting scar-related dissatisfaction, as previously reported by Grahn et al^3^ and Quinn and Wells.^33^

A systematic review^10^ reported a higher risk of nonunion in patients treated nonoperatively (72%) compared with those treated operatively (4%). Consistent with previous studies, nonunion was significantly more common in the patients treated with casting in the present cohort (2.7% vs 68.6%). However, nonunion was not associated with reduced patient-reported outcome measures or delayed return to athletic activities.

### Limitations

This study has some limitations. Despite the multicenter study design, the sample size remained limited; however, it met the statistical power calculation for the planned noninferiority trial.^17^ While the prespecified age-based subgroup analysis could not be conducted, all randomized patients completed the 12-month follow-up, and the inclusion of the declined cohort strengthens the generalizability of the findings. Future studies with extended recruitment periods or multinational collaboration may further explore age-related differences in treatment efficacy. An additional limitation of this study is that recruitment began before trial registration was posted on August 22, 2020, due to an oversight. However, the study followed a predefined protocol, received ethical approval beforehand, and adhered to CONSORT guidelines.

Blinding of the patients and their families to the treatment was not possible, potentially resulting in treatment bias.^34^ The study included only patients with isolated fractures, with or without initial elbow dislocation, who were able to engage in the study protocol; patients with more complex injuries were excluded. The estimates for the incidence of adverse events are subject to considerable uncertainty due to the relatively small sample size.

Randomization successfully balanced the baseline patient characteristics, and there was no crossover from the nonoperative cohort to the operative cohort. The QDASH questionnaire in Finnish has been validated for pediatric upper extremity fractures.^35^ Preinjury health-related quality of life was not recorded, as this cannot be reliably measured in children with an acute fracture. The minimal clinically important difference for the QDASH score has been estimated to be 6.8 for upper extremity injuries, and this difference was not observed at any follow-up time point between the study groups.^26^

## Conclusions

In this randomized clinical trial, casting without reduction was noninferior to open reduction and internal fixation in pediatric patients with displaced humeral medial epicondyle fractures. While this trial provides strong 1-year evidence, future studies should assess long-term outcomes into adolescence, particularly elbow function under load and late instability.

## Notes

Supplement 1.
Trial Protocol

Supplement 2. **eAppendix 1.** FiPO (Finnish Pediatric Orthopedics) Investigators and Roles **eAppendix 2.** Surgeons Level of Expertise and Instructions for Treatment and Follow-Up **eAppendix 3.** Mobilization Instructions Given to Patients After Cast Removal **eTable 1.** Reasons for Exclusion **eTable 2.** Information about Surgery **eTable 3.** Missing Data **eTable 4.** Outcome at 4 Weeks **eTable 5.** Outcome at 3 Months **eTable 6.** Outcome at 6 Months **eTable 7.** Baseline Demographics and Characteristics, Declined Cohort

Supplement 3. **Nonauthor Collaborators.** The Finnish Pediatric Orthopedics Investigators

Supplement 4.
Data Sharing Statement
