# Supplementary material for: Casting vs Surgical Treatment of Children With Medial Epicondyle Fractures: A Randomized Clinical Trial
Source: JAMA Netw Open. 2025 May 6;8(5):e258479. doi: 10.1001/jamanetworkopen.2025.8479 (PMC12056563; doi:10.1001/jamanetworkopen.2025.8479)
Supplement: Supplement 1. — Trial Protocol [file jamanetwopen-e258479-s001.pdf]

METHODS AND ANALYSIS

Study design:

The study is designed as a multicenter parallel-group non-inferiority randomized controlled trial (RCT) that complies with the CONSORT guidelines (Figure 1). A patient preference arm will be available. Patient recruitment will be done at all university hospital areas of Finland (Helsinki, Kuopio, Oulu, Tampere, Turku). The study is coordinated by Helsinki University Central Hospital, Children's Hospital pediatric orthopedic unit. Trial data analysts and person performing the recruitment will be unaware of the assigned treatment.

Patient recruitment:

All patients with a medial epicondyle fracture referred to the aforementioned hospitals will be screened for eligibility by a specialist of either hand surgery, pediatric surgery, pediatric orthopedics or orthopedics. If inclusion criteria are met, written consent is asked from the guardian. Patients and parents are given a written informed consent regarding the trial. The patient version is age adjusted for easier understanding according to the Finnish Investigators Network for Pediatric Medicines (www.finpedmed.fi)

Inclusion criteria:

Patients aged 7-16 years presenting with a ≥ 2mm displaced non-incarcerated medial epicondyle fracture with or without concomitant elbow dislocation and normal ulnar nerve function.

Exclusion criteria:

Pathological fracture, open fracture, systemic bone disease, concomitant fracture or injury of the same upper limb requiring operative intervention, other disease preventing participation in full follow-up regime and range of motion exercises.

Randomization

After agreeing to participation in the trial patients are randomized according to a computer-generated randomization list[16] to either operative or non-operative treatment. Randomization ratio is 1:1. Randomization is performed by the recruiting physician who is blinded to the intervention.

Patient's choice arm:

Patients who meet inclusion criteria, but refuse participation in the randomized trial are offered to choose treatment method (operative or non-operative) and continue in a prospective parallel patient preference arm that otherwise follow the same treatment and FU protocol as the RCT.

Baseline

Standard anterior-posterior and lateral radiographs of the elbow will be obtained after closed reduction of the possible elbow dislocation. All participants in either the RCT or patient's choice arm undergo cone-beam or normal computer tomography (CT) before treatment initiation. Initial fracture dislocation will be calculated from the CT scans in three planes (anterior-posterior, cranial-caudal and medial-lateral) and both radiographs (anterior-posterior and lateral) using the method described by Edmonds et al (2010)[17]. Date of injury, method of injury, patient's age at time of injury, sex, injured side, dominant hand and main sport or musical instrument as well as level will be documented. Motor and sensory function as well as range of motion of both upper limbs will be assessed. Carrying angle (degrees) and valgus stress test will be assessed if possible for both limbs.

Intervention

Non-operative treatment means upper limb immobilization with forearm in neutral pro-supination with a long arm cast for 4 weeks. Treatment is started after baseline examination.

Operative treatment is scheduled after baseline examination and is to be done within 7 days from injury. During sedation both elbows are stress tested using the valgus stress test, any instability is documented, carrying angle of both elbows are measured. Procedure of preference is open reduction and internal fixation (ORIF) with cannulated non-resolvable 4.0 mm screw with or without washer. If the fracture fragment is too small or fragmented for screw fixation 1.6 mm – 1.8 mm Kirshner-wires and/or bone anchor are used. After fracture fixation the injured side is again stress tested. Radiographs (anterior-posterior and lateral) documenting the fixation are taken. Long arm cast with forearm in neutral pro-supination is applied for 4 weeks. Time from injury to surgery, fixation method, length of surgery (min) and surgeon's level of training (consultant, registrar) will be documented. Hardware is not routinely removed.

All patients will receive a written exercise plan explaining the active and passive ROM exercises that are to be performed at a minimum three times per day from cast removal. Physiotherapy will be offered if guardians and/or patients feel that no progress in ROM after 2 weeks of home exercises.

Blinding

This trial tests a clinical intervention that is not suitable for protection against treatment bias. Recruiter will be blinded. Consultant on duty at will perform randomization and allocation. Non-operative treatment will be started immediately after recruitment. ORIF will be performed by surgeon on duty. Surgeon is not blinded. Trial data is collected at each appointment at the outpatient clinic by a physicians not related to the trial. Statistician analyzing trial data is blinded to treatment group.

Outcome measure

Follow-up is set at 1, 3, 6, 12 and 24 months from initiation of treatment with the option of ending the FU at 12 months if patients is pain free with full ROM in relation to uninjured side. Elbow standard radiograph (anterior-posterior and lateral) are taken at each appointment from 3 months on until bone union is achieved or trial ends (Table 1).

Patients will be examined at the pediatric orthopedic outpatient clinic. Upon each appointment active and passive ROM of both upper limbs (elbow extension-flexion, pro-supination, wrist extension-flexion) as well as carrying angle are measured using a goniometer. Stability of both elbows are assessed using the moving valgus test[18] and the valgus stress test[19]. Distal sensation is examined by Semmes-Weinstein monofilaments[20]. Signs of cold intolerance will be assessed. Grip strength is measured with a dynamometer.

Patients and guardians are requested to answer the following patient reported outcome measures at each appointment; QuickDASH©[21], Pediatric Quality of Life Inventory™ (PedsQL), PedsQL Pediatric Pain Questionnaire[22], cosmetic visual analogue scale (VAS 0-100) and Mayo elbow performance score (MEPS)[23].

Time of returning to main sport or music and its level will be documented (weeks). Any adverse effects (wound infection, nerve damage) are documented as well as hardware problems and possible hardware removal as well as conversion of treatment during FU (cast to ORIF or ligament reconstruction).

Primary outcome:

Statistically significant difference in QuickDASH score is 6.8 (18) at 12 months FU.

Secondary outcome:

Difference in active ROM in comparison to uninjured arm, PedsQL, PEDS QL Pain module, Cosmetic VAS, MEPS, need for additional procedures.

Sample size

Based on the results of Nikolas et al (2020)[24] and Aasheim et al (2014)[25] we assume clinically significant difference between the groups to be 6,8 and the standard deviation of the QuickDASH score to be 10

points. With 0,05 significance level and 80% power a non-inferiority comparison would require 27 patients per group. Allowing a 20% dropout rate the required sample would be 30 patients per group.

For subgroup analysis (less than 12 years vs. 12 years and over) 30 patients per age group needs to be collected. Assuming 50-50 split in the patients between the age groups the sample size would be 60 per ORIF and non-operated equaling a total of 120 patients.

Statistical analysis:

Data will be analyzed by using the Wilcoxon rank-sum test in Python 3.8. (Python Software Foundation, Wilmington, Delaware, U.S.A). Our hypothesis is that there is no difference in outcome between non-operative versus ORIF. Level of significance is set at $p < 0.05$.

Both treatment groups will be internally analyzed for differences in primary outcome regarding age (less than 12 years vs. 12 years and over) at time of injury and amount of initial fracture displacement (mm). Depending on group size, patient choice arm can be merged for analysis to same RCT group.

Ethics and dissemination:

There is no common consensus for dislocated (>2mm) medial epicondyle fractures. Treatment method vary by clinic and treating surgeon. Both ORIF and long arm cast are well established treatment methods for humeral medial epicondyle fractures. We have obtained ethical approval from Helsinki University Hospital (HUS) ethical board HUS/1443/2019. A permission to conduct the trial will be obtained by each study center. A written authorization from guardian will be acquired and child will be informed about the trial. Results of the trial will be disseminated as published articles in peer-reviewed journals.

Time schedule:

Last patient FU is expected by the end of 2023 and publication by the end of 2024.

CONCLUSION

The goal of this study is to compare two well-established treatment methods of dislocated non-incarcerated humeral medial epicondyle fractures in 7-16 year old patients.

Figure 1 Flow chart of the study

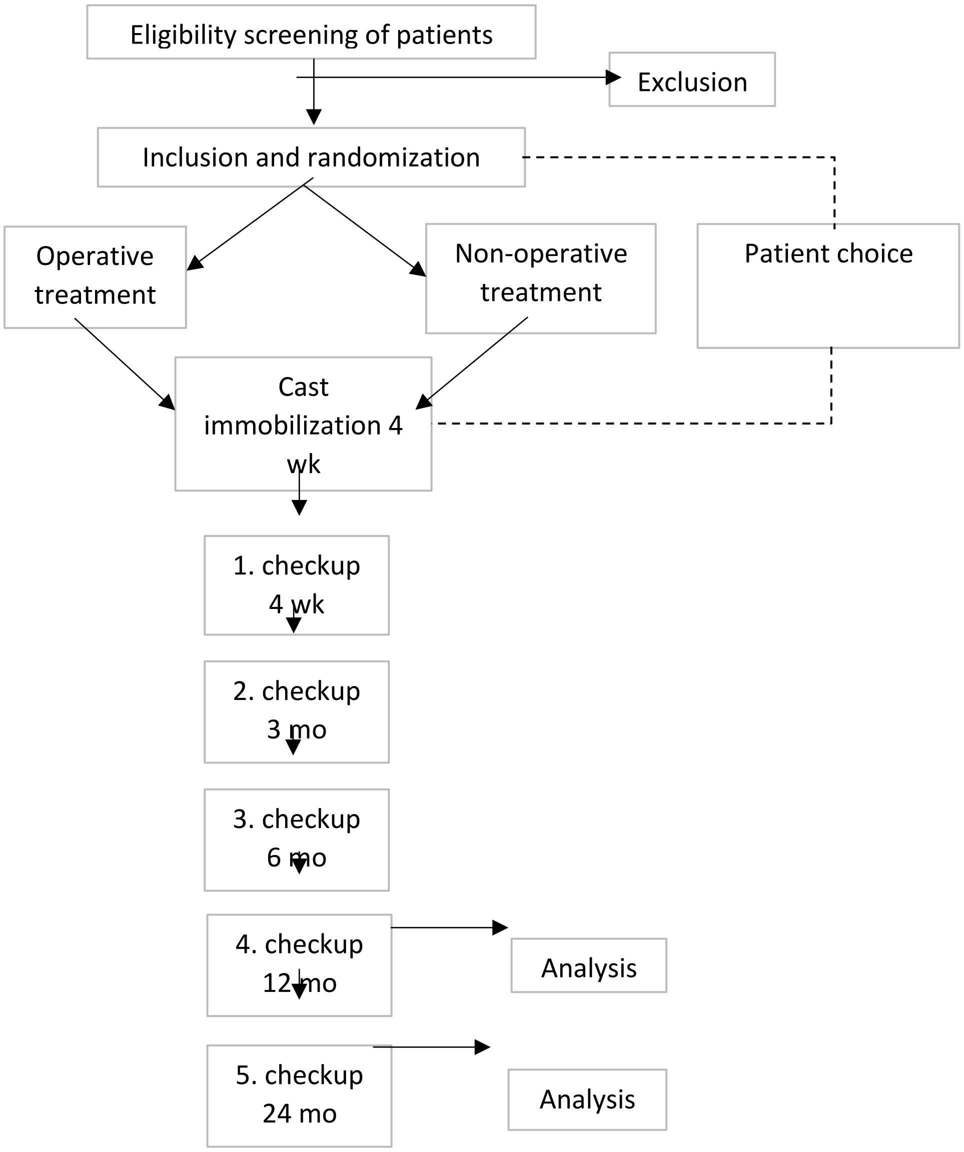

Table 1 **Data collection time points**

	Baseline	Treatment day 0	1. Checkup 1 mo	2. Checkup 3 mo	3. Checkup 6 mo	4. Checkup 12months	5. Checkup 24months
Diagnosis, eliqibility	x						
Randomisation	x						
Surgery or non-operative treatment		x					
Physical examination	x		x	x	x	x	x
Questionnaires			x	x	x	x	x
Computer tomography	x						
Standard radiograph	x		x	x	x	x	x
