# Supplementary material for: Casting vs Surgical Treatment of Children With Medial Epicondyle Fractures: A Randomized Clinical Trial
Source: JAMA Netw Open. 2025 May 6;8(5):e258479. doi: 10.1001/jamanetworkopen.2025.8479 (PMC12056563; doi:10.1001/jamanetworkopen.2025.8479)
Supplement: Supplement 2. — eAppendix 1. FiPO (Finnish Pediatric Orthopedics) Investigators and Roles eAppendix 2. Surgeons Level of Expertise and Instructions for Treatment and Follow-Up eAppendix 3. Mobilization Instructions Given to Patients After Cast Removal eTable 1. Reasons for Exclusion eTable 2. Information about Surgery eTable 3. Missing Data eTable 4. Outcome at 4 Weeks eTable 5. Outcome at 3 Months eTable 6. Outcome at 6 Months eTable 7. Baseline Demographics and Characteristics, Declined Cohort [file jamanetwopen-e258479-s002.pdf]

Supplementary Online Content

Grahn P, Helenius I, Hämäläinen T, et al; Finnish Pediatric Orthopedic Study Group Investigators. Casting vs surgical treatment of children with medial epicondyle fractures: a randomized clinical trial. *JAMA Netw Open.* 2025;8(5):e258479. doi:10.1001/jamanetworkopen.2025.8479

eAppendix 1. FiPO (Finnish Pediatric Orthopedics) Investigators and Roles

eAppendix 2. Surgeons Level of Expertise and Instructions for Treatment and Follow-Up

eTable 1. Reasons for Exclusion

eTable 2. Information about Surgery

eTable 3. Missing Data

eTable 4. Outcome at 4 Weeks

eTable 5. Outcome at 3 Months

eTable 6. Outcome at 6 Months

eTable 7. Baseline Demographics and Characteristics, Declined Cohort

eAppendix 3. Mobilization Instructions Given to Patients After Cast Removal

This supplementary material has been provided by the authors to give readers additional information about their work.

© 2025 Grahn P et al. *JAMA Network Open*.

eAppendix 1. FiPO (Finnish Pediatric Orthopedics) Investigators and Roles

Members of FiPo who apart from the authors have contributed to the study Casting vs. Surgical Treatment of Pediatric Medial Epicondyle Fractures: A Randomized Non-Inferiority Trial

Helsinki University Hospital

Juho-Antti Ahola, M.D.

Topi Laaksonen, M.D, Ph.D.

Kaj Zilliacus, M.D.

Turku University Hospital

Arimatias Raitio M.D, Ph.D.

Markus Lastikka M.D., Ph.D.

© 2025 Grahn P et al. *JAMA Network Open*.

Role of Authors and Contributors

Writing Committee

Petra Grahn, M.D., Ph.D., Principal Investigator, Chair, Helsinki University Hospital

Matti Ahonen, M.D., Ph.D., Principal Investigator, Co-chair, Helsinki University Hospital

Ilkka Helenius, M.D., Ph.D., Principal Investigator, Turku and Helsinki University Hospital

Yrjänä Nietosvaara, M.D., Ph.D., Principal Investigator, Kuopio and Helsinki University Hospital

Steering Committee

Petra Grahn

Matti Ahonen

Ilkka Helenius

Yrjänä Nietosvaara

Contributions

Study Concept and Design:

Petra Grahn M.D, PhD, Matti Ahonen M.D, PhD, Yrjänä Nietosvaara M.D. PhD, Ilkka Helenius M.D., PhD, Yrjänä Nietosvaara M.D., PhD, Tero Hämäläinen M.D.

Statistics:

Eliisa Löytyniemi, MSc,

Bishwesvar Singh

Data Management:

Zica Valendinos, Helsinki University Hospital

Elisa Wibom-Järvinen, Turku University Hospital

Patient Contacting:

Zica Valendinos, Helsinki University Hospital

Elisa Wibom-Järvinen, Turku University Hospital

© 2025 Grahn P et al. *JAMA Network Open*.

Site Investigators

Helsinki University Hospital

Petra Grahn, Matti Ahonen, Juho-Antti Ahola M.D., Topi Laaksonen M.D., Ph.D., Mikko Haara M.D., Ph.D., Mikko Mattila M.D., Ph.D., Kaj Zilliacus M.D.

Kuopio University Hospital

Yrjänä Nietosvaara, Jenni Jalkanen M.D., Ph.D.

Turku University Hospital

Ilkka Helenius, Markus Lastikka M.D., Ph.D., Arimatias Raitio M.D., Ph.D.

Oulu University Hospital

Juha-Jaakko Sinikumpu M.D., Ph.D

© 2025 Grahn P et al. *JAMA Network Open*.

eAppendix 2. Surgeons Level of Expertise and Instructions for Treatment and Follow-Up

Patient recruitment was conducted at four of the country's five university hospitals. All these trial centers serve as primary referral centers for major pediatric trauma. All surgeries were either performed or supervised by a specialist in pediatric orthopedics, orthopedics, hand surgery, or pediatric surgery, each with a minimum of three years of expertise. Instructions given to treating surgeons can be found below (Appendix Group A and B).

The clinical examination was performed by residents or specialists not related to the trial. They were all thought how to use a Jamar dynamometer, and a goniometer as well as how to perform the Valgus Stress and Moving Valgus tests and measure range of motion. Visual aid was also available for how to correctly mark flexion, extension, pro-supination and carrying angles.

GROUP A

Surgical Instructions for Patients in the Medial Epicondyle Study:

The surgery should be performed within 0-7 days from the injury.

The surgery is to be conducted according to AO guidelines (https://aotrauma.aofoundation.org/). The surgeon should review the guidelines in advance. Screw fixation (Asnis 4.0 or equivalent) is preferred. If the fragment is too small, fixation with two K-wires, as described in the AO guidelines, is used. The K-wire size should be either 1.4 or 1.6. The wound should be closed with absorbable sutures, such as Vicryl 4.0 or equivalent.

Navigate to AOtrauma.org -> AO Surgery References -> Pediatric -> Distal Humerus -> 13-M/7M Proceed -> Open Epicondylar Reduction and Internal Fixation -> Medial Approach -> Steps 1-4.

The surgeon decides whether to operate with the patient in the prone or supine position.

Antibiotics by surgeon’s choice, documented.

Patient is in general anesthesia in complete muscle relaxation. Tourniquet is used. The fixation material and patient positioning must be documented precisely in the surgical report.

GROUP B

Instructions for Casting Patients in the Medial Epicondyle Study:

The casting should be done on the day of the injury or at the latest on the next working day if the injury was initially unnoticed or if staff skilled in casting were not available.

© 2025 Grahn P et al. *JAMA Network Open*.

The casting is performed according to AO guidelines (https://aotrauma.aofoundation.org/). The surgeon should review the guidelines in advance.

Navigate to Aotrauma.org -> AO Surgery References -> Pediatric -> Distal Humerus -> 13-M/7M Proceed -> Splint Immobilization.

The surgeon decides on the casting material (fiberglass, traditional plaster, wood). The material choice is documented.

© 2025 Grahn P et al. *JAMA Network Open*.

eTable 1. Reasons for Exclusion

Supplementary Table 1. Reason for exclusion

	Number
Declined	19
Incarserated	10
Partial fracture	8
Other trauma affecting the same limb	6
Treament started elsewhere	5
Language barrier	2
Outside age limit	2
Ulnar nerve dysfunction	1
Psycho-social reasons	1
**All**	**54**

eTable 2. Information about Surgery

Supplementary table2[a]. Information about Surgery

	**Surgery (n=37)**	**Declined cohort (n=9)**
**Surgery time, mean (SD)[range], min**	47.3 (20.4) [14-111]	52.9 (15.5) [24-75]
**Use of profylactic antibiotics**	19 (51.4)	3 (33.3)
**Fixation method**		
Cannulated 4.0mm screw	36 (97.3)	8 (88.9)
Other (n)[b]	1	1
**Surgeons level of experience**		
Specialist	31 (83.8)	5 (55.6)
Resident (n)[c]	6	4

[a] Data are presented as number (percentage) of patients unless otherwise indicated.
[b] Other metod was 1 bone anchor in the Surgery group, and 1 Kirshner wire fixation in the Declined cohort.
[c] All resident surgeies were supervised by a specialist.

© 2025 Grahn P et al. *JAMA Network Open*.

eTable 3. Missing Data

Supplementary Table 3. Missing data

	Time point in trial											
	4 weeks			3 months[b]			6 months[b]			12 months		
**Item**	**Surgery**	**Cast**	**Declined**	**Surgery**	**Cast**	**Declined[c]**	**Surgery**	**Cast**	**Declined**	**Surgery**	**Cast**	**Declined**
QDASH	5	3	2	14	9	5	0	6	2	0	0	0
PedsQL	4	1	2	15	10	5	0	6	2	0	0	0
PedsQL Pain	3	1	2	13	10	4	0	6	1	0	0	0
MEPS	N/A	N/A	N/A	12	9	4	0	5	1	0	0	0
Cosmetic VAS	4	3	4	13	12	5	0	9	2	0	0	0
Clinical assessment[a]	1	0	0	12	10	3	0[d]	5	0	0	0	0
Radiographs	0	0	0	12	9	2	0	5	0	0	0	0

[a] Includes range of motion, carrying angle, elbow stability assessment, grip strength, sensory assesment.
[b] Due to retriction on the health care casued by the COVID pandemic the 3 and 6 month time points were skipped if the patients were healing as expected. The COVID restrictions were in place 16.3-15.6.2020 and 1.3-27.4.2021. In addition a large nurses strike prohibited recruitment and follow- up of patients 20.4.2022-4.5.2022.
[c] 1 patient lost to follow up.
[d] 2 patients are missing moving valgus, and valgus stress data.

© 2025 Grahn P et al. *JAMA Network Open*.

eTable 4. Outcome at 4 Weeks

Supplementary Table 4. Quick Disabilities of Arm, Shoulder and Hand Score (QDASH) and Secondary Outcomes at 4 weeks

	Mean, Median (Q1, Q3)		
**Outcome**	**Surgery (n=36)**	**Casting (n=35)**	**p-value**
**Primary outcome**			
QDASH score	31.6, 29.6 (17.1, 42.1)	34.6, 35.0 (20.4, 43.2)	.25
95% CI	25.2 to 37.9	28.5 to 40.7	
**Secondary outcomes**			
**Clinical findings**			
Elbow range of motion[a]	47.1, 45.0 (32.5, 63.5)	56.9, 55.0 (40.0, 72.5)	.08
Flexion deficit[b]	43.5, 43.5 (36.0, 50.0)	34.5, 32.0 (28.0, 41.0)	.004
Extension deficit[b]	56.0, 60 (44.0, 71.0)	54.4, 55.0 (40.0, 67.5)	.02
Carrying angle deficit[c]	NA	NA	NA
Moving valgus test, (%)[c]	NA	NA	NA
Valgus stress test, (%)[c]	NA	NA	NA
Grip strength[d]	8.3, 8.0 (6.0, 10.0)	7.0, 6.0 (4.5, 7.9)	.04
**QDASH module score**			
Sports or performing arts	78.5, 93.7 (69.0, 100.0)	86.6, 100.0 (75.0, 100.0)	.46
**PedsQL[e]**			
Total score	74.7, 79.3 (67.0, 83.9)	74.5, 74.0 (68.0, 83,0)	.64
Physical function score	63.0, 65.6 (50.0, 78.0)	61.9, 59.4 (53.0, 68.8)	.48
**[illegible]QL Pain[f]**			
Current pain	9.1, 4.2 (1.1, 9.0)	8.1, 2.1 (0.0, 8.5)	.28
Worst pain in last 7 days	21.1, 7.7 (2.0, 44.2)	14.05, 3.7 (0.0, 16.0)	.15
**Cosmetic VAS[g]**	63.3, 70.2 (46.0, 82.0)	63.5, 70.0 (48.0, 86.8)	.95
**[illegible][h]**	67.7, 65.0 (40.0, 65.0)	71.76, 77.5 (55.0, 85.0)	.38

Abbrevations: QDASH, Quick Disabilities of Arm, Shoulder and Hand; Q1 and Q3, Upper (Q1) and Lower (Q3) quater of the interquartile range; NA, Not applicable.

[a] Measured by goniometer and reported as the difference in degrees between full flexion and extension

[b] Elbow flexion, extension and carrying angle deficit as compared to the uninjured side and reported as difference in degrees

[c] Measurments were not conducted due to elbow extenson deficiency following cast treatment

[d] Measured with a dynamometer, and expressed as difference to uninjured side (kg)

[e] Pediatric Quality of Life (PedsQL) is a brief measure of health-related quality of life in children and young people with a subscale describing the physical function. Both are scored from 0 (worst possible) to 100 (best possible).

[f] Ranges from 0 (no pain) to 100 (worst imaginable pain)

[g] Ranges from 0 (worst possible appearance) to 100 (best possible appearance)

[h] The Mayo Elbow Preformance Score (MEPS) is a composite score out of 100 used to report elbow outcome with higher scores reflecting better results

© 2025 Grahn P et al. *JAMA Network Open*.

eTable 5. Outcome at 3 Months

Supplementary Table 5. Quick Disabilities of Arm, Shoulder and Hand Score (QDASH) and Secondary Outcomes at 3 months

	Mean, Median (Q1, Q3)		
**Outcome**	**Surgery (n=25)**	**Casting (n=25)**	**p-value**
**Primary outcome**			
QDASH score	3.9, 2.3 (0.0, 6.8)	7.4, 4.0 (0.0, 11.4)	.67
95% CI	1.7 to 6.2	2.9 to 11.9	
**Secondary outcomes**			
**Clinical findings**			
Elbow range of motion[a]	135.2, 140.0 (130.0, 145.0)	125.0, 125.0 (116.0, 140.0)	.02
Flexion deficit[b]	2.3, 0.0 (0.0, 5.0)	5.8, 4.0 (0.0, 10.0)	.11
Extension deficit[b]	6.3, 5.0 (0.0, 10.0)	11.0, 5.0 (-3.0, 20.0)	.38
Carrying angle deficit[b]	-1.3, 0.0 (0.0, 0.0)	-2.0, 0.0 (-4.0, 0.0)	.42
Moving valgus test, (%)[c]	1 (3.8)	5 (16.7)	.19
Valgus stress test, (%)[c]	2 (7.7)	7 (28.0)	.07
Grip strength[d]	0.9, 1.0 (-0.5, 3.0)	2.1, 2.0 (1.0, 4.0)	.13
**QDASH module score**			
Sports or performing arts	14.5, 0.0 (0.0, 12.5)	8.5, 0.0 (0.0, 12.5)	.86
**PedsQL[e]**			
Total score	90.8, 91.7 (85.0, 96.3)	85.6, 88.0 (80.4, 94.7)	.24
Physical function score	90.4, 93.9 (84.0, 100.0)	87.0, 91.0 (81.2, 94)	.46
**PedsQL Pain[f]**			
Current pain	2.2, 0.0 (0.0, 2.1)	6.2, 0.0 (0.0, 3.2)	.88
Worst pain in last 7 days	7.9, 1.1 (0.0, 9.7)	12.7, 0.0 (0.0, 13.0)	.95
**Cosmetic VAS[g]**	83.5, 89.0 (78.7, 100.0)	84.9, 88.8 (80.0, 100.0)	.96
**MEPS[h]**	95.6, 100.0 (100.0, 100.0)	92.7, 100.0 (85.0, 100.0)	.15

Abbrevations: QDASH, Quick Disabilities of Arm,Shoulder and Hand; Q1 and Q3, Upper (Q1) and Lower (Q3) quater of the interquartile range.

[a] Measured by goniometer and reported as the difference in degrees between full flexion and extension

[b] Elbow flexion, extension and carrying angle deficit as compared to the uninjured side and reported as difference in degrees

[c] Number of patients with instability as compared with uninjured side. In the valgus stress test, the examiner reports, either pain, loosness or both. Surgery group; 2 pain. Cast group; 1 pain, 3 loosness, 4 both to uninjured side

[d] Measured with a dynamometer, and expressed as difference to uninjured side (kg)

[e] Pediatric Quality of Life (PedsQL) is a brief measure of health-related quality of life in children and young people with a subscale describing the physical function. Both are scored from 0 (worst possible) to 100 (best possible).

[f] Ranges from 0 (no pain) to 100 (worst imaginable pain)

[g] Ranges from 0 (worst possible appearance) to 100 (best possible appearance)

[h] The Mayo Elbow Preformance Score (MEPS) is a composite score out of 100 used to report elbow outcome with higher scores reflecting better results

© 2025 Grahn P et al. *JAMA Network Open*.

Supplementary Table 6. Quick Disabilities of Arm, Shoulder and Hand Score (QDASH) and Secondary Outcomes at 6 months			
	Mean, Median (Q1, Q3)		
**Outcome**	**Surgery (n=37)**	**Casting (n=30)**	**p-value**
**Primary outcome**			
QDASH score	3.7, 0.0 (0.0, 6.8)	4.2, 0.0 (0.0, 4.5)	.84
95% CI	1.6 to 0.0	1.4 to 0.0	
**Secondary outcomes**			
**Clinical findings**			
Elbow range of motion[a]	144.1, 145.5 (137.5, 145.5)	135.3, 137.0 (130, 145)	.01
Flexion deficit[b]	2.8, 0.0 (0.0, 5.0)	4.9, 0.0 (0.0, 8.0)	.14
Extension deficit[b]	2.4, 0.0 (0.0, 5.0)	7.1, 1.0 (0.0, 10.0)	.10
Carrying angle deficit[b]	-1.1, 0.0 (-1.5, 0.0)	-0.1, 0.0 (0.0, 0.0)	.54
Moving valgus test, (%)[c]	2 (5.7)	4 (13.3)	.40
Valgus stress test, (%)[c]	3 (8.6)	9 (30.0)	.05
Grip strength[d]	0.6, 0.0 (-1.0, 2.0)	0.8, 1.0 (0.0, 2.0)	.68
**QDASH module score**			
Sports or performing arts	5.5, 0.0 (0.0, 0.0)	7.2, 0.0 (0.0, 6.0)	.63
**PedsQL[e]**			
Total score	91.6, 93.3 (86.5, 99.0)	91.0, 92.0 (87.0, 96.3)	.69
Physical function score	93.3, 95.5 (91.0, 100.0)	92.5, 94.0 (88.0, 97.0)	.68
**PedsQL Pain[f]**			
Current pain	1.7, 0.0 (0.0, 2.1)	1.9, 0.0 (0.0, 1.1)	.36
Worst pain in last 7 days	7.3, 2.1 (0.0, 8.4)	6.0, 0.0, (0.0 11.2)	.47
**Cosmetic VAS[g]**	76.1, 84.3 (70.9, 94.8)	86.2, 100.0 (85.0, 100.0)	.01
**MEPS[h]**	96.7, 100.0 (100.0, 100.0)	95.5, 100.0 (95.0, 100.0)	.26

Abbrevations: QDASH, Quick Disabilities of Arm,Shoulder and Hand; Q1 and Q3, Upper (Q1) and Lower (Q3) quater of the interquartile range.

[a] Measured by goniometer and reported as the difference in degrees between full flexion and extension

[b] Elbow flexion, extension and carrying angle deficit as compared to the uninjured side and reported as difference in degrees

[c] Number of patients with instability as compared with uninjured side. In the valgus stress test, the examiner reports, either pain, loosness or both. Surgery group; 2 pain, 1 loosness. Cast group; 3 pain, 2 loosness, 4 both compared to the uninjured side.

[d] Measured with a dynamometer, and expressed as difference to uninjured side (kg)

[e] Pediatric Quality of Life (PedsQL) is a brief measure of health-related quality of life in children and young people with a subscale describing the physical function. Both are scored from 0 (worst possible) to 100 (best possible).

[f] Ranges from 0 (no pain) to 100 (worst imaginable pain)

[g] Ranges from 0 (worst possible appearance) to 100 (best possible appearance)

[h] The Mayo Elbow Preformance Score (MEPS) is a composite score out of 100 used to report elbow outcome with higher scores reflecting better results

© 2025 Grahn P et al. *JAMA Network Open*.

Supplementary Table 7. Baseline Demographics and Characteristics, Declined Cohort[a]

Characteristics	Surgery (n=9)	Casting (n=12)
Age at injury, mean (SD)[range], y	12.4 (2.2) [9.6-15.4]	12.5 (2.2) [7.0-15.2]
**Sex**		
Female	5 (55.6)	7 (58.3)
Male	4	5
**Dominant side injured**	4 (44.4)	4 (33.3)
**Injury mechanism[b]**		
Gymnastics	6 (66.7)	6 (50)
Arm wrestling/wrestling	1	0
Fall from standing hight	2	3
Fall from height	0	3
**Elbow dislocation[c]**	4 (44.4)	5 (41.7)
**Primary fracture dislocation, mean (SD) [range], mm[d]**		
Antero-posterior radiograph	8.4 (3.8) [6-14]	8.7 (2.8) [5.0-13.0]
Lateral radiograph	8.1 (2.3) [6-13]	8.1 (7.1) [1-18]
Coronal computed tomography	7.0 (73.3) [3-13]	6.8 (3.2) [3-13.5]
Sagittal computed tomography	12.0 (2.6) [9-15}	11.6 (4.4) [2-17]
**Time from injury to treatment, mean (SD) [range], d**	3 (2.5) [0-8]	1.2 (1.0) [0-3]
**Immobilization time, mean (SD) [range], w**	4.3 (0.3) [3.9-4.6]	4.2 (0.5) [3.5-5.1]

Abbrevations: y, years; mm, millimeters; d, days; w, weeks.
[a] Data are presented as number (percentage) of patients unless otherwise indicated.
[b] Gymnastics also includes cheerleading, parkour, bouldering, and dancing. Fall from standing heights also include ball games (soccer and ice hockey). Fall from heigts also include biking, electric scooter, downhill skiing, skateboarding.
[c] Radiographic complete elbow joint dislocation.
[d] Displacement was measured from radiographs and computed tomography images as described by Edmonds et al[18].

eAppendix 3. Mobilization Instructions Given to Patients After Cast Removal (Translation at the End)

FRACTURE OF THE MEDIAL EPICONDYLE

After removing the cast or sling, you can start using your arm as usual! However, sports involving contact or other activities that could lead to falls on the injured arm should be avoided for the first few weeks. Once your arm is pain-free and you can touch your shoulder with your fingertips and fully straighten your elbow, there are no longer any restrictions on physical activity.

The exercises below will help you restore movement in your elbow joint. You can do these exercises at home on your own or with a parent, both in the morning and evening. You can't do these exercises too much! Remember, you must exercise your arm yourself, as no one else can do it for you! A little pain associated with the exercises is not dangerous.

Regaining movement may take time. Generally, the mobility of the elbow joint returns to normal within two months after removing the cast.

Do each of the following exercises for at least 3 minutes 3 times per day.

1. **Stretching with a Pillow**
   Lie on your back and place your elbow on a pillow with your palm facing the ceiling. Relax the upper limb and allow it to extend as much as possible. If desired, you can hold a light weight in your hand or ask a parent to help straighten the arm.

2. **Elbow Flexion**
   Sit down with your arm extended as straight as possible alongside your body. Begin by bending your arm at the elbow as much as possible. Then, use your healthy hand to gently apply pressure to bring your fingers closer to your shoulder. After this, lower your arm back to its original straight position and repeat.

3. **Hanging**
   Position yourself on a climbing frame or rings so that your feet touch the ground. Place your hands as high as possible on the climbing frame and bend your knees until you feel a stretch in your elbow. If you wish and it does not cause too much pain, you can hang freely.

Translation to English for JAMA

© 2025 Grahn P et al. *JAMA Network Open*.
