# Supplementary material for: Casting vs Surgical Treatment of Children With Medial Epicondyle Fractures: A Randomized Clinical Trial
Source: JAMA Netw Open. 2025 May 6;8(5):e258479. doi: 10.1001/jamanetworkopen.2025.8479 (PMC12056563; doi:10.1001/jamanetworkopen.2025.8479)
Supplement: Supplement 3. — Nonauthor Collaborators. The Finnish Pediatric Orthopedics Investigators [file jamanetwopen-e258479-s003.pdf]

Supplemental Online Content: Nonauthor Collaborators
*First name, last name, and suffix (if applicable) are required and will appear in PubMed.

*Group Name(s): FiPO (Finnish Pediatric Orthopedics) Investigators							
***First Name and Middle Initial(s)**	***Last Name**	***Suffix (eg, Jr, III)**	Academic Degrees	Institution	Location (city, state/province, country)	Role or Contribution, eg, chair, principal investigator	Group (if more than 1 Group listed in the byline) and/or Subgroup (eg, Steering Committee)
Juho-Antti	Ahola		M.D.	Department of Pediatric O	Helsinki, Finland	Site investigator	
Topi	Laaksonen		M.D., Ph.D.	Department of Pediatric O	Helsinki, Finland	Site investigator	
Kaj	Zilliacus		M.D.	Department of Pediatric O	Helsinki, Finland	Site investigator	
Arimatias	Raitio		M.D., Ph.D.	Department of Pediatric Su	Turku, Finland	Site investigator	
Markus	Lastikka		M.D., Ph.D.	Department of Pediatric Su	Turku, Finland	Site investigator	
