# Supplementary material for: Casting vs Surgical Treatment of Children With Medial Epicondyle Fractures: A Randomized Clinical Trial
Source: JAMA Netw Open. 2025 May 6;8(5):e258479. doi: 10.1001/jamanetworkopen.2025.8479 (PMC12056563; doi:10.1001/jamanetworkopen.2025.8479)
Supplement: Supplement 4. — Data Sharing Statement [file jamanetwopen-e258479-s004.pdf]

Data Sharing Statement

Grahn. Casting vs Surgical Treatment of Children With Medial Epicondyle Fractures. *JAMA Netw Open*. Published May 06, 2025. doi:10.1001/jamanetworkopen.2025.8479

Data
Additional Information: ClinicalTrials.gov Identifier: NCT04531085.
Data available: Yes
Data types: Deidentified participant data
How to access data: Data will be provided upon request to other researchers in the field provided the request fullfills the EU GDPR laws and an IRB for the study has been approved
When available: With publication

Supporting Documents
Document types: Other (please specify)
Additional Information: If forementioned data is requested itwill be supplied. However the information is all in Finnish or Swedish
How to access documents: corresponding author: petra.grahn@hus.fi
When available: With publication

Additional Information
Who can access the data: Data will be provided upon request to other researchers in the field provided the request fullfills the EU GDPR laws and an IRB for the study has been approved
Types of analyses: Only for research purposes
Mechanisms of data availability: Data will be provided upon request to other researchers in the field provided the request fullfills the EU GDPR laws and an IRB for the study has been approved. Also a signed data access agreement will be needed
Any additional restrictions: none
